# “Stewards of the future: accompanying the rising tide of young voices by setting youth-inclusive research agendas in sustainability research”

**DOI:** 10.1186/s42055-021-00041-w

**Published:** 2021-02-10

**Authors:** Alicia Donnellan Barraclough, Melina Sakiyama, Lisen Schultz, Inger Elisabeth Måren

**Affiliations:** 1 Department of Biological Sciences, University of Bergen, Bergen, Norway; 2 UNESCO Chair on Sustainable development and Environmental Management, University of Bergen, Bergen, Norway; 3 Youth Voices Programme, Global Youth Biodiversity Network & Biodiversity Programme, Forum für Umwelt und Entwicklung, Berlin, Germany; 4 Stockholm Resilience Centre, Stockholm University, Stockholm, Sweden; 5 Centre for Sustainable Area Management, University of Bergen, Bergen, Norway

## Abstract

**Background:**

From the worldwide youth-led climate strikes of 2018–2019 to the student-initiated austerity protests in Chile in 2019, it is undeniable that young people have been protagonists in pressuring for social change towards greater sustainability in recent years. This piece reflects on youth as agents of social-ecological change, and what researchers can learn from the rise in youth-led social movements demanding action in the face of global sustainability challenges.

The study of sustainability problems like climate change and biodiversity loss, intrinsically requires consideration of inter-generational equity. However, despite 50% of the global population being under 30 years old, youth are often not included explicitly as actors in environmental social sciences and sustainability-related research. Here we discuss why explicitly considering young people as distinct actors during the research process is important, as it allows researchers to engage in just and inclusive work whilst at the same time accounting for important agents of change in complex social-ecological systems.

**Results:**

As a framework for our inquiry we present the themes which emerged during a series of international meetings and forums on sustainability challenges and youth in 2019, a year characterized by world-wide youth mobilization. Our briefing spans the United Nations Youth 2019 Climate Action Summit, the post-2020 meetings organized by the youth branch of the Convention of Biological Diversity (CBD), the UNESCO Man and Biosphere Youth forums and the results from the Global Shapers Survey of the World Economic Forum.

**Conclusions:**

We argue that if researchers wish to facilitate youth-inclusive evidence-based decision making, research agendas must address knowledge gaps highlighted by institutional efforts to incorporate youth concerns within global sustainability policy, a recommendation that is even more relevant in the light of the COVID-19 crisis. We draw on the themes which emerged in our analysis of international youth meetings to provide recommendations for research agendas which account both for young actors as both passive and active components of social-ecological change and we propose a more inclusive and holistic study of coupled natural-human systems.

## Background: The rising tide of young voices in times of planetary crisis

In the public sphere, there is a growing global momentum behind social movements led by youth that demand bold action in the face of inequality, climate change and biodiversity decline. As a show of commitment to the younger generations, the United Nations (UN) Secretary General called the first ever UN Youth Climate Action Summit in New York in September 2019. This meeting was one example of several efforts by international governing bodies to explicitly include youth perspectives into international policy agendas on societal transitions, another notable example being the wave of youth consultations underway to inform the post-2020 biodiversity framework of the Convention on Biological Diversity (CBD).

Research dealing with coupled economic, social and ecological systems is advancing interdisciplinary and transdisciplinary approaches to address the major sustainability challenges of the Anthropocene [1, 2]. Guided by international policy agendas and frameworks, such as the Sustainable Development Goals of the Agenda 2030 [3]*,* transdisciplinary research on sustainability is exploring avenues for incremental and transformative change needed to achieve fair and equitable sustainable development across the globe whilst tackling the biodiversity and climate crises [1]. However, the explicit treatment of young people as stakeholders that are shaping and being shaped by social-ecological systems in the Anthropocene is still rare in sustainability literature, with some exceptions [4]^.^ Here we reflect on youth as key agents of social-ecological change and present the emerging themes from youth-led social movements and international forums for youth voices. We then use these themes as a basis to reflect on how the research community can account for the perspectives of younger generations in their research.

## We need to set youth sensitive and youth inclusive research agendas to accompany global policy

Youth are a group of special focus in sustainability policy, and feature in numerous SDGs; education (SDG4), job security (SDG9), reduction of inequalities (SDG11) and Climate Action (SDG14), as a part of the “leave no one behind” framework [5]. Several SDGs make a call to increase participation and capacity building of all at risk groups, including youth, and to “Ensure responsive, inclusive, participatory and representative decision-making at all levels” [3] . Broadening inclusion and participation are thus seen as a transformational goal key to accomplishing the Agenda 2030 [1]. Multilateral environmental agreements, such the Convention of Biological Diversity (CBD), also contain clauses specific to increasing youth participation in all areas of environmental governance (COP11 Decision XI/9) [6].

Firstly, if we are to understand the effects of sustainability-related policy implementation and guarantee evidence-based decision making, research agendas and policy frameworks must go hand in hand. Therefore, research should help facilitate a fuller understanding of the impacts of global change and sustainable development “wicked problems” on youth specifically [7–9]. Secondly, research into sustainability challenges requires reflexivity, and as researchers we must constantly examine the relationship between our research and the broader society [10]. Despite the existence of multiple frameworks within the sustainability research community to engage in socially responsible research, including tools for multi-stakeholder involvement [11], the topic of youth-inclusive research has received very little attention in the equity and sustainability debate [8, 12]. A Web of Science search of the terms sustain* and youth (or young*) yields 0 hits in leading journals such as Nature or Science. Although pockets of literature exist within the environmental social science literature which address the vulnerability of younger generations to specific global sustainability challenges [4, 7, 9, 13, 14], a collective recognition of the need to explicitly put youth on the research agenda is lacking.

Lastly, the study of social-ecological transformation and sustainability transitions specifically might benefit from internationally targeting youth in research, a fact which has become ever more relevant since the *COVID-19* pandemic. Not only are youth potentially impactful agents of social-ecological change through their influence in social movements [15, 16], but also though driving changes in the economic system through changing values or consumption patterns [17]. Therefore, understanding the views, needs and concerns of youth in the face of global change should become an explicit research priority in fields concerned with the environment, equity and social justice.

## A sea of youth concerns

**Fig. 1:**
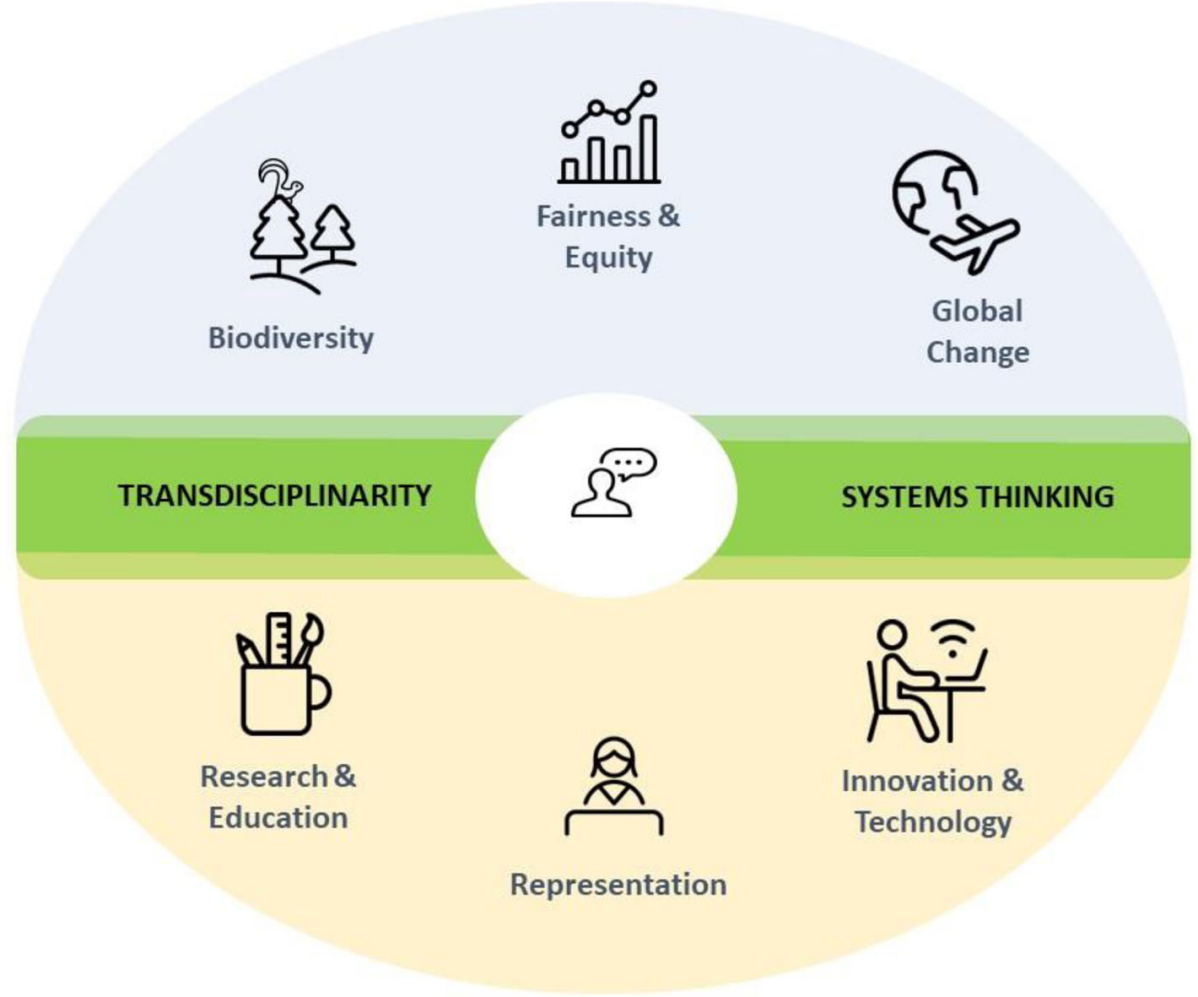
Major themes emerging from international youth forums (UN Youth Climate Action Summit and UNESCO Man and Biosphere and CBD post-2131 forum) and the Global Shapers Survey by the World Economic Forum. The green band across the circle denotes two key concepts underpinning the six themes: transdisciplinarity and systems thinking

Young people’s emerging concerns on the major sustainability challenges of the Anthropocene should provide a thematic roadmap to guide environmental sustainability and social-ecological transformation researchers (Fig. 1). Here we present our account of the major themes arising in international meetings for youth voices, such as the UN Youth Climate Action Summit (https://www.un.org/en/climatechange/youth-in-action) and the UN post-2030 CBD youth forums. We support our conclusions with the results of the recent Global Shapers Survey (GSS) conducted by the World Economic Forum (*n* = 30,000) [18].

### Youth and global change

The GSS showed that “climate change/destruction of nature” was ranked as the most serious global issue with 49.9% of votes, a trend uniform across age groups [18]. Numerous youth-led initiatives supported by international institutions aim to tackle and raise awareness of the environmental crisis and climate change, including the UN Summer of Solutions, the UN Climate Reboot Troops, the UNESCO Man and Biosphere (MAB) youth network, and the Global Youth Biodiversity Network (GBYN). Grassroots movements have recently drawn global attention to the climate crisis, with youth climate strike mobilization statistics hitting an all-time high in September 2019, with an estimated 6 million strikers worldwide lead by youth organizations.

### Youth, nature and biodiversity

Destruction of nature is ranked first as a global youth concern [18]. Youth have worked to be represented within major multilateral environmental agreements such as the CBD. In 2012, GYBN advocated to include a youth-specific clause in the Conference of the Parties meeting in Hyderabad (COP11) [19]. This clause promotes youth involvement in all stages of the planning and the implementation of national biodiversity strategies (COP11 Decision XI/9). Youth declarations published across youth networks [19] speak of the need to address the biodiversity and the climate crisis as one systemic issue, calling for transformative change which holds nature-based solutions at its centre.

### Youth, social justice, fairness and equity

The GSS showed that “Large-scale conflict/wars” and “inequality (income, discrimination)” were ranked as the second and third most important global issues, with 39.10 and 30.9% of votes, respectively [18]. Youth demand that implementation of sustainability agendas must guarantee equitable and fair benefit sharing and be sensitive to the global south context. Sustainability initiatives must also empower and engage local communities and Indigenous peoples in all phases of planning and implementation. Additionally, there are calls for the mainstreaming of gender and minority equity, diversity and inclusivity within all sectors involved in sustainability policy planning and implementation [19].

### Youth, representation and power

The GSS ranked “government accountability and transparency/corruption” as a primary concern within their own countries (46.10% of votes) [18]. Multiple youth forums in 2019 stressed the lack of political will to implement concrete and transformative actions needed to address sustainability crises. There is also a clear call across youth organizations to increase representation of young stakeholders with capacity to influence policy and decision making. According to a 2019 study by the Inter-parliamentary Union, only 2.2% of members of parliament (MPs) are below the age of 30 worldwide, and a push to increase representation is seen as central to promoting youth decision making capacity and influence worldwide [20].

### Youth, innovation and technology

In addition to deeper systemic approaches, youth have been protagonist of innovation and technology-based solutions to tackle global sustainability challenges. Notable initiatives like the “Summer of Solutions” and the “Climate Reboot Troops”, under the auspices of the UN Envoy on Youth, have kick-started youth-led projects using open data and tech concepts to solve a local environmental crisis unique to each location and community [21]. Youth discussions have also urged caution in this sector, pointing out that techno-fix pathways should not be prioritized as “silver-bullet” solutions to sustainability challenges, opting for deeper systemic approaches with economic, social and ecological transformation at their centre [19].

### Youth, research and education

Education and outreach have been raised as central to achieving sustainability outcomes, stressing the need to fund and implement capacitation, upskilling and awareness raising schemes across the globe. Youth reflect a need for an education and research agenda that contemplates diverse knowledge systems, that promotes critical thinking and addresses power asymmetries. Additionally, youth call for an increase in cross-sectorial and interdisciplinarity platforms, which allow for emergent transdisciplinary collaborations and approaches to sustainability challenges. In the academic context, more than 80% of youth in the GSS disagreed with the statement “Academics and scientific experts should not be involved in politics” [18], reflecting a need to close divides between sectorial silos, facilitate evidence-based policy and address the research-implementation gap.

Although we have presented these six themes as separate, underlying them is a holistic mindset underpinned by two key concepts: systems thinking and transdisciplinary (Fig. 1). In our personal experience facilitating youth forums, millennials are very comfortable dealing with overlapping layers of complexity, systems and networks. In the minds of many youth the environmental crisis cannot be separated from key drivers like inequality or poor governance. Therefore, youth movements are demanding from world leaders a mindset that can follow these complexities, and can provide perhaps not a solution, but an integrated “crisis-management” roadmap that aims for and is fully committed to systemic change. Youth are demanding that these systemic changes address the core of our development models, including the values and principles which underpin them. In the same way, transdisciplinary and cross-sectorial efforts must create new languages and approaches [22], which are able to address the connections between inequality and unequal distribution of resources, concentration of power, failing governance systems and institutions, and the unprecedented environmental degradation, poverty and risks to young people and future generations.

Last but not least, despite the simplistic presentation of these themes, it is important to acknowledge that youth are not a uniform group, and that Global North narratives have traditionally dominated global youth discourse [8, 18]. Thus, the importance of any of these concerns is strongly dependent on socio-economic and cultural context, which is in itself a research gap which needs addressing [8].

## Future directions in setting inter-generational youth-inclusive research agendas and practices in sustainability research

Researchers studying coupled social-ecological systems and sustainability, particularly those working in emerging disciplines within sustainability science, such as the the environmental social sciences (e.g. socioecology, ecological economics and political ecology), should labor to produce research which accounts for the role of younger generations in shaping the past, present and future of social-ecological systems. There are multiple avenues for scientific research to foster incremental and transformative social learning opportunities which account for youth, and here we point out three simple and complementary pathways:

### Practice inclusion

Remembering youth as essential stakeholders and utilizing appropriate participatory and social learning methods are key steps to producing youth-inclusive research. Potential practices include: (a) Involve youth in participatory processes such as horizon scanning [23], participatory scenario building [24], future-casting [25] and dialogue [26] processes which capture youth’s concerns and contribute to collaborative research agendas. (b) Explicitly recognize youth more broadly as key stakeholders in conservation and sustainability projects, including them in the study sample as well as in planning, consultations, mapping exercises, and community follow-ups and monitoring. (c) Foster collaborations and empower young sustainability leaders from both academic and practitioner backgrounds, promoting youth-led initiatives and questions [14]. (d) Engage in responsible research practices following guidelines such as the Responsible Research and Innovation framework [27]. Ensuring fair multi-stakeholder initiatives also means remaining sensitive to local and historical contexts, being age and gender responsive, and aware of socio-cultural issues, including the role of power dynamics in knowledge coproduction [28].

### Address knowledge gaps

In order to identify and address knowledge gaps we must: (a) Combine research synthesis with participatory and multi-stakeholder engagement processes in order to increase our baseline knowledge of the specific roles of youth as agents of change in social-ecological transformation [29] (b) Enhance the use of existing methods which capture the complex nature of youth as a both a passive and active agent in sustainability issues. For example, employing transdisciplinary methods used to study complex system dynamics [29], such as: participatory action research, participatory scenario planning, participatory games, participant observation and dialogue workshops. (c) Advance new youth-friendly methodologies for capturing youth concerns and perspectives in sustainability research. This requires cross-sectorial and transdisciplinary collaborations, which facilitate method development that builds on existing techniques from the health and social sciences, utilizing emerging technologies when appropriate [30].

### Facilitate engagement and build trust

(a) Foster platforms for building trust and understanding *(8)*. Although the GSS showed that academic and research institutions were among the most trusted institutions by youth, continuing to work on building understanding and trust between the research community and younger generations is essential. (b) Facilitate youth-targeted science outreach and collaborative education programmes, essential to building an understanding amongst the younger generations of the role of science in a changing world. (c) Encourage alternative research outputs, which reach broader audiences and are accessible to younger generations. (d) Encourage reflexivity during the whole research process [10]. Create discussion spaces for self-evaluation where researchers can reflect and discuss on the impact of their research on the broader community, and facilitate learning from failure [31].

To conclude, we call for action to accompany youth-inclusive sustainability policy with youth-inclusive research agendas. Thus, we hope researchers will foster inter-generational, multi-stakeholder and multidisciplinary forums that allow knowledge exchange, and that can function as opportunities for researchers to become sensitive to the needs and perspectives of youth. By doing so, we build the trust and understanding needed to move forward in setting research agendas which recognize the role of youth in our common future.

## Data Availability

Data used for the classification of concerns is freely available at the World Economic Forum website for “Global Shapers Survey”.

## References

[1] Sachs JD, Schmidt-Traub G, Mazzucato M, Messner D, Nakicenovic N, Rockström J. Nat Sustain. 2019;2:805–14.

[2] Kates RW, Clark WC, Corell R, Hall JM, Jaeger CC, Lowe I, McCarthy JJ, Schellnhuber HJ, Bolin B, Dickson NM, Faucheux S, Gallopin GC, Grubler A, Huntley B,Jager J, Jodha NS, Kasperson RE, Mabogunje A, Matson P, Mooney H, Moore B, O’Riordan T, Svedin U. Environment and development - Sustainability science. Science. 2001;292(5517):641–2. 10.1126/science.1059386 11330321

[3] UN General Assembly, Transforming our world: the 2030 Agenda for Sustainable Development. 2015, A/RES/70/1, available at: https://www.refworld.org/docid/57b6e3e44.html.

[4] Gallay E, Pykett A, Smallwood M, Flanagan C. Flanagan. Urban youth preserving the environmental commons: student learning in place-based stewardship education as citizen scientists. Sustainable Earth. 2020;3(1):3.

[5] United Nations. Work Stat. Comm. Pertain. to 2030 Agenda. Sustain Dev. 2019:1–21.

[6] CBD, COP 11 Decision XI/6 XI/6. Cooperation with Other Conventions, International Organizations, and Initiatives, 2012.

[7] Ruesga-Benito SM, González-Laxe F, Picatoste X. Sustainable development, poverty, and risk of exclusion for young people in the European Union: The case of NEETs. Sustainability (Switzerland). 2018;10(12):1–15.

[8] Walker C. Uneven solidarity: the school strikes for climate in global and intergenerational perspective. Sustainable Earth. 2020;3(1):5.

[9] Sanson AV, Van Hoorn J, Burke SE. Responding to the Impacts of the Climate Crisis on Children and Youth. Child Development Perspectives. 2019;13(4):201–7.

[10] Knaggård Å, Ness B, Harnesk D. Finding an academic space: Reflexivity among sustainability researchers. Ecology and Society. 2018;23(4).

[11] Mauser W, Klepper G, Rice M, Schmalzbauer BS, Hackmann H, Leemans R, Moore H. Transdisciplinary global change research: The co-creation of knowledge for sustainability. Current Opinion in Environmental Sustainability. 2013;5(3–4):420–31.

[12] Leach M, Reyers B, Bai X, Brondizio ES, Cook C, Díaz S, Espindola G, Scobie M, Stafford-Smith M, Subramanian SM. Equity and sustainability in the Anthropocene: a social–ecological systems perspective on their intertwined futures. Global Sustainability. 2018;1.

[13] Rekola A, Paloniemi R. Researcher-planner dialogue on environmental justice and its knowledges-a means to encourage social learning towards sustainability. Sustainability (Switzerland). 2018;10(8).

[14] Gordon IJ, Bawa K, Bammer G, Boone C, Dunne J, Hart D, Hellmann J, Miller A, New M, Ometto J, Pickett S, Wendorf G, Agrawal A, Bertsch P, Campbell CD, Dodd P, Janetos A, Mallee H, Taylor K. Forging future organizational leaders for sustainability science. Nature Sustainability. 2019;2(8):647–9.

[15] Pelenc J, Wallenborn G, Milanesi J, Sébastien L, Vastenaekels J, Lajarthe F, Ballet J, Cervera-Marzal M, Carimentrand A, Merveille N, Frère B. Alternative and Resistance Movements: The Two Faces of Sustainability Transformations? Ecological Economics 159(December 2018). 2019:373–8.

[16] Sievers-Glotzbach S, Tschersich J. Overcoming the process-structure divide in conceptions of Social-Ecological Transformation: Assessing the transformative character and impact of change processes. Ecological Economics 164 (December 2018). 2019:106361.

[17] Godelnik R. Millennials and the sharing economy: Lessons from a ‘buy nothing new, share everything month’ project. Environmental Innovation and Societal Transitions. 2017;23:40–52.

[18] WEF, Global Shapers Annual Survey, 2017.

[19] G.Y.B. Network, (n.d.).

[20] I. UNION. Youth Participation in National Parliaments; 2018. p. 2014.

[21] United Nations. Technology and Innovation Labs, (2019).

[22] Lang DJ, Wiek A, Bergmann M, Stauffacher M, Martens P, Moll P, Swilling M, Thomas CJ. Transdisciplinary research in sustainability science: Practice, principles, and challenges. Sustainability Science 2012;7(SUPPL. 1):25–43.

[23] Sutherland W, Woodroof H. The need for environmental horizon scanning Articulo Revisión. Trends Ecol Evol. 2009. 10.1016/j.tree.2009.04.008 19660827

[24] Oteros-rozas E, Martín-lópez B, Daw TM, Bohensky EL, Butler JRA, Hill R, Martin J. Participatory Scenario Planning in Place-Based Social-Ecological Research: Insights and Experiences from 23 Case Studies; 2015.

[25] Pereira LM, Hichert T, Hamann M, Preiser R, Biggs R. Using futures methods to create transformative spaces: visions of a good Anthropocene in southern Africa. 2018;23(1).

[26] Abma TA, Broerse JEW. Patient participation as dialogue: setting research agendas. Health Expectations. 2010;13(2):160–73. 10.1111/j.1369-7625.2009.00549.x PMC5060528 20536537

[27] Consortium RT. A practical guide to responsible research and innovation; n.d.

[28] Tengö M, Brondizio ES, Elmqvist T, Malmer P, Spierenburg M. Connecting diverse knowledge systems for enhanced ecosystem governance: the multiple evidence base approach. Ambio. 2014;43(5):579–91. 10.1007/s13280-014-0501-3 PMC4132468 24659474

[29] Preiser R, Biggs R, De Vos A, Folke C. Social-ecological systems as complex adaptive systems: Organizing principles for advancing research methods and approaches. Ecology and Society. 2018;23(4).

[30] Filho WL, Marans RW, Callewaert J. Handbook of sustainability and social science research. Cham: Springer International Publishing; 2018.

[31] Catalano AS, Redford K, Margoluis R, Knight AT. Black swans, cognition, and the power of learning from failure. Conservation Biology 2018;32(3):584–596. 10.1111/cobi.13045 29094402

